# Psychometric Evaluation of the Internalized Stigma of Mental Illness Scale for Patients with Mental Illnesses: Measurement Invariance across Time

**DOI:** 10.1371/journal.pone.0098767

**Published:** 2014-06-02

**Authors:** Chih-Cheng Chang, Tsung-Hsien Wu, Chih-Yin Chen, Jung-Der Wang, Chung-Ying Lin

**Affiliations:** 1 Department of Psychiatry, Chi Mei Medical Center, Tainan, Taiwan; 2 Health Service and Population Research Department, Institute of Psychiatry, King's College London, London, United Kingdom; 3 Department of Senior Citizen Service Management, College of Recreation and Health Management, Chia Nan University of Pharmacy and Science, Tainan, Taiwan; 4 Department of Psychiatry, Chi Mei Medical Center, Liouying, Tainan, Taiwan; 5 Department of Nursing, College of Health Sciences, Chang Jung Christian University, Tainan, Taiwan; 6 Deparment of Public Health, College of Medicine, National Chen Kung University, Tainan, Taiwan; 7 Departments of Internal Medicine and Occupational and Environmental Medicine, National Cheng Kung University Hospital, Tainan, Taiwan; United (Osaka U, Kanazawa U, Hamamatsu U Sch Med, Chiba U and Fukui U) Graduate School of Child Development, Japan

## Abstract

**Background:**

The current investigation examined the psychometric properties of the Internalized Stigma of Mental Illness (ISMI) scale in a sample of patients with mental illness. In addition to the internal consistency, test-retest reliability, and concurrent validity that previous studies have tested for the ISMI, we extended the evaluation to its construct validity and measurement invariance using confirmatory factor analysis (CFA).

**Methods:**

Three hundred forty-seven participants completed two questionnaires (i.e., the ISMI and the Depression and Somatic Symptoms Scale [DSSS]), and 162 filled out the ISMI again after 50.23±31.18 days.

**Results:**

The results of this study confirmed the frame structure of the ISMI; however, the Stigma Resistance subscale in the ISMI seemed weak. In addition, internal consistency, test-retest reliability, and concurrent validity were all satisfactory for all subscales and the total score of the ISMI, except for Stigma Resistance (α = 0.66; ICC = 0.52, and *r* = 0.02 to 0.06 with DSSS). Therefore, we hypothesize that Stigma Resistance is a new concept rather than a concept in internalized stigma. The acceptable fit indices supported the measurement invariance of the ISMI across time, and suggested that people with mental illness interpret the ISMI items the same at different times.

**Conclusion:**

The clinical implication of our finding is that clinicians, when they design interventions, may want to use the valid and reliable ISMI without the Stigma Resistance subscale to evaluate the internalized stigma of people with mental illness.

## Introduction

The stigma of mental illness, unlike the stigma of other medical conditions (e.g., epilepsy, leprosy, and cancer), is still highly prevalent in most high-income countries with good healthcare [1], [2]. People with a mental illness are easily discriminated against because of the negative stereotypes attached to mental illness [3]. Therefore, the stigma they face erodes their social status, interpersonal relationships, quality of life, and self-esteem [4]–[6]. As a result, people with mental illness are at a high risk of unemployment, isolation, and delayed treatment-seeking, which often causes a serious public health burden.

One aspect of the stigma is internalized stigma (self-stigma), discussion of which has increased over the past few decades [2]. Because people with mental illness encounter external and objective discrimination, such as reduced access to employment and housing, they may translate this discrimination into self-devaluation. Thus, people with mental illness are left feeling that they are not members of the society in which they live [7]. In addition, even those who have not experienced discrimination (the behavioral manifestation of public stigma) may also feel alienated because of prejudice (the attitudinal manifestation of public stigma) [8]. Ritsher (Boyd) et al. [7] call this kind of stigma “internalized stigma”, and say that it is “one of the especially painful and destructive effects of stigma”. Based on their definition, Ritsher (Boyd) et al. [7] developed a reliable and valid instrument, the Internalized Stigma of Mental Illness (ISMI) scale for people with mental illness, and the ISMI has been broadly used in different cultures [9].

The ISMI is useful for clinicians, and this validated measure encourages researchers and clinicians to use, in addition to symptom reduction, self-stigma reduction as a concurrent treatment goal [7]. Link et al. [10] reported that stigma works at cross-purposes to treatment, and Ritsher (Boyd) et al. [7] said that interventions that reduce internalized stigma and illness symptoms are efficient and long lasting.

The ISMI was validated in 2003, and the psychometric properties of the original version have been tested [7]. The Turkish version [11], the Korean version [12], the German version [13], and the original version, which was adapted for people with leprosy [14], have been tested. All five studies in the previous sentence reported that the ISMI has high internal consistency (α>0.90) and good test-retest reliability (*r* = 0.62–0.92), and that it is reliable and valid. However, to the best of our knowledge, no other studies have evaluated the reliability and validity of the ISMI for versions in other languages. Although Boyd et al. [9] did a comprehensive ISMI review that provided useful information about the psychometric properties of the multinational versions of ISMI, only five of the studies they reviewed [7], [11], [12], [14], [15] said that evaluating the psychometric properties of the ISMI was an objective of their study. In addition, one recent review [16] on internalized stigma scales found that only three studies—Ersoy & Varan [11], Rensen et al. [14], and Ritsher (Boyd) et al. [7]—evaluated the reliability and validity of the ISMI, which indirectly means that the psychometric evaluation of the ISMI is insufficient.

In addition to the insufficient analysis of the psychometric properties of the multinational versions of the ISMI, the construct validity of the ISMI is still under development. Stevelink et al. [16] claim that adequate factor analysis has never been applied to the psychometric properties of ISMI, which resulted in an “indeterminate rating”. After reviewing the five studies on the ISMI's psychometric properties [7], [11]–[14], we tentatively concluded that the ISMI has not been examined for its construct or theoretical frame structure.

Another important question about the construct and the item descriptions of the ISMI is whether they can be invariant across time. People with a mental illness may have cognitive difficulties that prevent them from being able to answer some items [17]. If, because of their cognitive dysfunction, people with mental illness are unable to answer some items on the ISMI, they may differently interpret the items as well as the construct of the ISMI across time. In other words, if the measurement invariance of the ISMI is not supported, it may not be stable enough to measure internalized stigma for people with mental illness under the construct of the ISMI. Because no studies have examined the construct of the ISMI or the measurement invariance of the ISMI, they both require evaluation. Specifically, the measurement invariance of the ISMI can test the equivalence of the means of its factor structure, factor loadings, item intercepts, and construct across time [18], and indicate whether mentally ill people with certain kinds of cognitive dysfunction interpret the ISMI the same across time.

The purposes of this study were (1) to establish three basic psychometric properties—internal consistency, test-retest reliability, and concurrent validity—of the ISMI Taiwan version, (2) to examine the theoretical construct of the ISMI, and (3) to test the measurement invariance of the ISMI across time.

## Methods

### Ethics statement, participants, and procedures

The study was approved by the Research and Ethics Review Board of the Chi Mei Medical Center (IRB number: 10102-L06). All of the participants were psychiatric outpatients, inpatients of psychiatric acute wards, psychiatric daycare patients, or psychiatric patients receiving homecare services from Chi Mei Medical Center. The inclusion criteria were (1) older than 20 years; (2) the ability to read, speak, and understand spoken Mandarin Chinese or Taiwanese; and (3) voluntary agreement to participate after the study purposes had been explained to them. Patients with unstable mental symptoms during the survey were excluded (i.e., patients were excluded if their psychiatric symptoms made them unable to complete the ISMI). In addition, each participant filled out a written informed consent.

Several psychiatrists first explained the purposes of the study to the recruited patients; 350 patients agreed to participate and signed the informed consents. Afterward, several research assistants asked each participant to fill out three questionnaires: the Internalized Stigma of Mental Illness (ISMI) scale, the Depression and Somatic Symptoms Scale (DSSS), and one background information sheet. Two weeks to 3 months later, using convenience sampling, 162 of the 350 participants were asked to complete the ISMI again.

### Instruments

#### Internalized Stigma of Mental Illness (ISMI) scale

The ISMI is a self-rated 29-item questionnaire with five subscales (Alienation, Stereotype Endorsement, Discrimination Experience, Social Withdrawal, and Stigma Resistance). Each item of the ISMI asks the respondents to express, using a 4-point Likert scale, how much they agree with the description. The ISMI has been validated since 2003, and the internal consistency and test-retest are acceptable for the original version (α = 0.72–0.90, test-retest *r* = 0.68–0.92); the Stigma Resistance subscale, however, has an α of 0.58 [7]. In addition, the concurrent validity of the ISMI has been examined using the correlation between the ISMI and other stigma-related concepts: the ISMI has been positively associated with devaluation-discrimination and depressive symptoms (*r* = 0.35 and 0.53, respectively), and negatively correlated with self-esteem (*r* = −0.59), empowerment (*r* = −0.52), personal empowerment (*r* = −0.34), and recovery orientation (*r* = −0.49) [7].

Although the Taiwan version of the ISMI has been translated [19], some terms used in the Taiwan version are apparently “politically correct” replacements (e.g., “mental illness” was replaced with “disability”), which makes the Taiwan version difficult to precisely measure for people with a mental illness. Therefore, we asked the ISMI developer, Prof. Boyd, for and were given permission to revise these troublesome terms in the Taiwan version. Two psychiatrists (the first author of this study, and one psychiatrist in the Psychiatry Department, Chung-Ho Memorial Hospital, Kaohsiung Medical University) made the lexical changes (See Table S1).

#### Depression and Somatic Symptoms Scale (DSSS)

The DSSS is a self-rated 22-item questionnaire with two domains (Depression domain: 12 items; Somatic domain: 10 items). The internal consistency (α = 0.73–0.94), test-retest reliability (*r* = 0.88–0.92), and convergent validity (*r* = 0.63–0.86 with the Hamilton Depression Rating Scale) are satisfactory. In addition, known-group validity has been established for the DSSS [20].

### Data analysis

In addition to the descriptive analyses for demographics and ISMI scores, Cronbach's α for internal consistency, intraclass correlation coefficients (ICC) for test-retest reliability, Pearson correlation coefficients (*r*) for concurrent validity, confirmatory factor analyses (CFAs), and multi-group CFAs in a repeated-measures design for measurement invariance were done.

An α>0.7 and an ICC value >0.75 suggest satisfactory internal consistency and test-retest reliability, respectively [21]. However, the test-retest interval in this study varied from 2 weeks to 3 months, was slightly long, and may have influenced the test-retest reliability. Therefore, we decided to additionally examine the test-retest reliability at intervals of <30 days (n = 75) and of <60 days (n = 116). For concurrent validity, the ISMI was assumed to be moderately correlated with the DSSS, and an *r*>0.3 was expected [22].

We used first-order and second-order models to test the structural frames of the ISMI, and used CFA with maximum-likelihood estimations. The first-order model had six subscales of ISMI correlated with each other, while the second-order model had the six subscales embedded in the second-order construct of the ISMI. In addition to a χ^2^ difference test (nonsignificant), we used comparative fit index (CFI) >0.95, root mean square of approximation (RMSEA) <0.08, and SRMR (standardized root mean square residual) <0.08 tests to examine the data-model fit [23].

Based on one review study of measurement invariance [24], we used test-retest data (n = 162), and four nested models were compared to test the invariance. The four models were as follows:

- Model 1: configural model;
- Model 2: model with factor loadings constrained as equal across time;
- Model 3: model with factor loadings and item intercepts constrained as equal across time;
- Model 4: model with factor loadings, item intercepts, and construct means constrained as equal across time.

We separately tested the measurement invariance for the subscales of Alienation, Stereotype Endorsement, Discrimination Experience, Social Withdrawal, and Stigma Resistance, because using all items and five subscales may violate the principle of parsimony for CFA. In addition, the ISMI construct across time was examined using the sum of the scores of the subscales as observed-item scores. Therefore, the ISMI construct contained first- and second-time ISMI scores as two correlated latent constructs, and the sums of 5 first-time subscale scores correlated with the sums of their second-time scores as 10 observed-item scores.

A nonsignificant χ^2^ difference test suggests measurement invariance; however, because χ^2^ is too sensitive to detect non-invariance in a large sample [25], we used the other three indices of ΔCFI, ΔRMSEA, and ΔSRMR. The recommendations of invariance are as follows: (1) ΔCFI>−0.01 with ΔRMSEA<0.015 for factor loadings, item intercepts, and construct means; (2) ΔSRMR<0.03 for factor loadings and <0.01 for item intercepts [26], [27].

All the demographic data, reliability, and concurrent validity were analyzed using SPSS 16.0 (SPSS Inc., Chicago, IL, USA). CFA and multi-group CFA were done using LISREL 8.8 (Scientific Software International, Lincolnwood, IL, USA).

## Results

### Demographics and ISMI scores

Because 3 participants did not answer all the questions on the ISMI, all their data were excluded from analysis; only the data from the other 347 participants were analyzed. The mean (± SD) age was 43.76±11.27 years; the mean age at onset was 31.88±11.81 years for the whole sample; and the mean ages for the test-retest sample (n = 162) were 43.20±10.29 and 30.00±10.19 years, respectively. There were 148 men and 199 women; 247 (70.9%) of the participants had a senior high school education or higher, and 156 (45%) were single. In addition, the test-retest sample completed the ISMI again with an interval of 50.23±31.18 days (Table 1).

**Table 1:** Demographic data of participants.

Characteristics	Whole sample (n = 347)		Test-retest sample (n = 162)	
	Mean or (n)	SD or %	Mean or (n)	SD or %
Age (years)	43.76	11.27	43.20	10.29
Age at onset (years)	31.88	11.81	30.00	10.19
Days between test-retest	—	—	50.23	31.18
Gender				
Male	(148)	42.7%	(74)	45.7%
Female	(199)	57.3%	(88)	54.3%
Education				
Junior high or less	(100)	28.8%	(38)	23.5%
Senior high	(146)	42.1%	(64)	39.5%
College or higher	(101)	28.8%	(60)	37.0%
Marital status				
Married	(138)	39.8%	(49)	30.2%
Single	(156)	45.0%	(90)	55.6%
Other	(53)	15.2%	(23)	14.2%
Diagnoses				
Schizophrenia & other psychosis	(161)	45.1%	(94)	58.0%
Depressive disorder	(98)	28.2%	(38)	23.5%
Bipolar disorder	(43)	12.4%	(21)	13.0%
Anxiety disorder	(33)	9.5%	(9)	5.6%
Other	(12)	3.5%	(0)	0.0%

The mean (± SD) ISMI scores were as follow: Alienation (A) = 2.11±0.74, Stereotype Endorsement (SE) = 1.98±0.63, Discrimination Experience (DE) = 2.08±0.70, Social Withdrawal (SW) = 2.15±0.72, and Stigma Resistance (SR) = 2.68±0.54.

### Reliability and concurrent validity

The entire 29-item ISMI had a satisfactory internal consistency (Cronbach's α = 0.94), and an excellent test-retest reliability coefficient (ICC = 0.78). The α and ICC values for the subscales were 0.89 and 0.79 for A, 0.86 and 0.75 for SE, 0.85 and 0.75 for DE, 0.89 and 0.80 for SW, and 0.66 and 0.52 for SR. In addition, ICC = 0.87 for A, 0.85 for SE, 0.84 for DE, 0.87 for SW, and 0.69 for SR with a test-retest interval <30 days; ICC = 0.82 for A, 0.76 for SE, 0.74 for DE, 0.81 for SW, and 0.55 for SR with an interval <60 days.

The concurrent validity analysis showed that 4 of the 5 ISMI subscales were significantly positively correlated with the Somatic (*r* = 0.42 for A, 0.39 for SE, 0.38 for DE, and 0.44 for SW; *p*s<0.001) and the Depression (*r* = 0.55 for A, 0.49 for SE, 0.46 for DE, and 0.55 for SW; *p*s<0.001) domains of the DSSS. However, the Stigma Resistance subscale was correlated with neither the Somatic (*r* = 0.06; *p* = 0.26) nor the Depression (*r* = 0.02; *p* = 0.71) domain.

### Confirmatory factor analysis and measurement invariance across time

Based on the theoretical construct, first- and second-order models were used for CFA. All fit indices for both were acceptable, except for the χ^2^ tests (first-order χ^2^ = 930.663; second-order χ^2^ = 936.299; *p*s<0.001). In addition, all the factor loadings were significant, except for the second-order factor of Stigma Resistance (Table 2).

**Table 2:** Factor loadings and data-model fit of Internalized Stigma of Mental Illness.

	1st-order	2nd-order
**Standardized factor loading**		
*Alienation*	—	*0.959*
I feel out of place in the world because I have a mental illness	0.713	0.711
Having a mental illness has spoiled my life	0.834	0.833
People without mental illness could not possibly understand me	0.753	0.754
I am embarrassed or ashamed that I have a mental illness	0.749	0.748
I am disappointed in myself for having a mental illness	0.864	0.864
I feel inferior to others who don't have a mental illness	0.680	0.681
*Stereotype Endorsement*	—	*0.982*
Stereotypes about the mentally ill apply to me	0.693	0.692
People can tell that I have a mental illness by the way I look	0.759	0.759
Mentally ill people tend to be violent	0.514	0.514
Because I have a mental illness, I need others to make most decisions for me	0.690	0.688
People with mental illness cannot live a good, rewarding life	0.752	0.752
Mentally ill people shouldn't get married	0.626	0.626
I can't contribute anything to society because I have a mental illness	0.755	0.756
*Discrimination Experience*	—	*0.992*
People discriminate against me because I have a mental illness	0.749	0.747
Others think that I can't achieve much in life because I have a mental illness	0.743	0.744
People ignore me or take me less seriously just because I have a mental illness	0.828	0.827
People often patronize me, or treat me like a child, just because I have a mental illness	0.574	0.574
Nobody would be interested in getting close to me because I have a mental illness	0.782	0.784
*Social Withdrawal*	—	*0.984*
I don't talk about myself much because I don't want to burden others with my mental illness	0.629	0.625
I don't socialize as much as I used to because my mental illness might make me look or behave ‘weird’	0.796	0.794
Negative stereotypes about mental illness keep me isolated from the ‘normal’ world	0.816	0.818
I stay away from social situations in order to protect my family or friends from embarrassment	0.821	0.823
Being around people who don't have a mental illness makes me feel out of place or inadequate	0.787	0.787
I avoid getting close to people who don't have a mental illness to avoid rejection	0.700	0.700
*Stigma Resistance*	—	*−0.107* a
I feel comfortable being seen in public with an obviously mentally ill person	0.193	0.191
In general, I am able to live life the way I want to	0.747	0.742
I can have a good, fulfilling life, despite my mental illness	0.808	0.815
People with mental illness make important contributions to society	0.428	0.424
Living with mental illness has made me a tough survivor	0.468	0.468
**Fit indices**		
χ^2^	930.663	936.299
*df*	367	372
*p*-value	<0.001	<0.001
CFI	0.979	0.979
RMSEA	0.068	0.068
SRMR	0.073	0.074

*df*  =  degree of freedom; CFI  =  comparative fit index; RMSEA  =  root mean square of approximation; SRMR  =  standardized root mean square residual; Stigma Resistance items are reversely coded; Second-order factor loadings are in *italics*.

a *p*  =  0.094; all other *p*s < 0.05 for factor loadings.

Four nested models were used to test the invariance of the 5 ISMI subscales. The full ISMI and all 5 subscales had acceptable configural models, and all were appropriate for further invariance testing.

Except for two χ^2^ difference tests (Model 3 vs. 2 for ISMI: χ^2^ = 13.705, *p*<0.05; Model 4 vs. 3 for SE: χ^2^ = 4.230, *p*<0.05), the fit indices of the ISMI and all its subscales supported measurement invariance across time (Table 3).

**Table 3:** Data-model fit indices and model comparisons for Internalized Stigma of Mental Illness (ISMI) and its five subscales (n = 162).

						Model					
Model #	χ^2^	*df*	CFI	RMSEA	SRMR	Comparisons	Δχ^2^	Δ*df*	ΔCFI	ΔRMSEA	ΔSRMR
**ISMI** a											
M1	56.387*	29	0.988	0.075	0.048	—	—	—	—	—	—
M2	61.907*	33	0.987	0.074	0.055	M2-M1	5.520	4	−0.001	−0.001	0.007
M3	75.612*	37	0.983	0.081	0.057	M3-M2	13.705*	4	−0.004	0.007	0.001
M4	77.426*	38	0.982	0.081	0.057	M4-M3	1.814	1	−0.001	0.000	0.000
**Alienation**											
M1	79.340*	47	0.988	0.067	0.046	—	—	—	—	—	—
M2	81.552*	52	0.989	0.061	0.051	M2-M1	2.212	5	0.001	−0.006	0.005
M3	90.060*	57	0.988	0.061	0.050	M3-M2	8.508	5	−0.001	0.001	0.000
M4	90.065*	58	0.988	0.060	0.050	M4-M3	0.005	1	0.000	−0.001	0.000
**Stereotype Endorsement**											
M1	118.136*	69	0.976	0.061	0.058	—					
M2	125.575*	75	0.976	0.059	0.065	M2-M1	7.439	6	0.000	−0.002	0.007
M3	132.194*	81	0.975	0.057	0.065	M3-M2	6.619	6	−0.001	−0.002	0.000
M4	136.424*	82	0.974	0.059	0.065	M4-M3	4.230*	1	−0.001	0.002	0.000
**Discrimination Experience**											
M1	36.621	29	0.995	0.036	0.042	—	—	—	—	—	—
M2	42.486	33	0.994	0.041	0.048	M2-M1	5.865	4	−0.001	0.005	0.006
M3	43.972	37	0.995	0.033	0.048	M3-M2	1.486	4	0.001	−0.008	0.000
M4	43.973	38	0.996	0.030	0.048	M4-M3	0.001	1	0.001	−0.003	0.000
**Social Withdrawal**											
M1	64.535*	47	0.994	0.047	0.040	—	—	—	—	—	—
M2	70.093*	52	0.993	0.045	0.058	M2-M1	5.558	5	−0.001	−0.002	0.019
M3	74.576	57	0.994	0.042	0.058	M3-M2	4.483	5	0.001	−0.003	0.000
M4	76.862*	58	0.993	0.043	0.058	M4-M3	2.286	1	−0.001	0.001	0.000
**Stigma Resistance**											
M1	61.352*	29	0.931	0.078	0.064	—	—	—	—	—	—
M2	63.281*	33	0.935	0.069	0.065	M2-M1	1.929	4	0.004	−0.008	0.001
M3	66.977*	37	0.936	0.065	0.065	M3-M2	3.696	4	0.001	−0.005	0.000
M4	69.412*	38	0.933	0.065	0.065	M4-M3	2.435	1	−0.003	0.001	0.000

*df*  =  degree of freedom; CFI  =  comparative fit index; RMSEA  =  root mean square of approximation; SRMR  =  standardized root mean square residual.

M1: Configural model; M2: All factor loadings were invariant between test and retest; M3: All factor loadings and item intercepts were invariant between test and retest; M4: All factor loadings, item intercepts, and construct means were invariant between test and retest.

a Internalized Stigma of Mental Illness: using the sum scores of the five subscales as observed item scores.

*p<0.05.

## Discussion

This is, to the best of our knowledge, the first examination of the construct of the ISMI that uses several CFA models to analyze its measurement invariance across time. We found that the data-model fit was acceptable for both the first- and the second-order CFA models, which suggests that the construct of the ISMI is appropriate. However, the factor loading of the Stigma Resistance subscale of the ISMI was very low (−0.107) and not significant; therefore, this may be a weak subscale. Moreover, internal consistency, test-retest reliability, and concurrent validity were satisfactory for the ISMI, except for the Stigma Resistance subscale. All the fit indices were acceptable for measurement invariance, and indicated that people with mental illness can interpret the ISMI the same across time.

Our internal consistency and test-retest reliability results were comparable to previous findings [7], [11]–[14]: the Cronbach's α of the ISMI total score are all >0.90. However, they are not 100% comparable because they are calculated using different test-retest methods: we used ICC, but Ritsher (Boyd) et al. [7] used Pearson correlation (*r*). As a result, our acceptable test-retest reliability coefficient for the total score of the ISMI (ICC = 0.78) seems slightly lower than the original version (*r* = 0.92). Pearson correlation does not detect any system errors [28] and may easily overestimate the real test-retest coefficient. Therefore, the ICC, which deals with the systematic bias, may yield lower coefficients than does the Pearson correlation [29]. Another reason may because of the test-retest interval [9]: for our study, it was 50.23±31.18 days, longer than the 42 days in Ritsher (Boyd) et al. [7]; thus, lower test-retest reliability might be acceptable.

All ISMI subscales but Stigma Resistance had satisfactory internal consistency and test-retest reliability. The low internal consistency of Stigma Resistance was also found in Ritsher (Boyd) et al. [7], and they suggested that it be reexamined. We therefore used CFA to confirm its low internal consistency, and found various factor loadings (about 0.75 for two items, 0.4 for two items, and 0.19 for one item) in our CFA models. Furthermore, our second-order CFA indicates that the construct of Stigma Resistance fit poorly in the ISMI framework.

Brohan et al. [8] suggested that although the ISMI measures internalized stigma, Stigma Resistance may not appropriately fit in the concept of internalized stigma, and our results support that opinion. In addition, in a 14-country study across Europe, Brohan et al. [30] found that Stigma Resistance was not a significant independent predictor for internalized stigma. Moreover, Sibitz et al. [6], [31] consider Stigma Resistance a new concept, and suggest developing a new instrument for Stigma Resistance. Therefore, we would suggest omitting the Stigma Resistance subscale if the researchers and clinicians focus only on measuring the internalized stigma of people with mental illness.

There are several possible explanations of why the Stigma Resistance subscale in the ISMI is weak. First, the items on that subscale are positively worded, but the items on the other subscales are negatively worded. Previous studies on the effect of wording suggest that using both negatively and positively worded items may bias the evaluation of the extracted constructs of instruments [32], [33]. Second, the subject of the five items of Stigma Resistance is inconsistent: four items use “I” and one uses “People with mental illness”. Third, based on our CFA factor loading results, we concluded that two items describe daily living (“In general, I am able to live life the way I want to” and “I can have a good, fulfilling life despite my mental illness”), two items describe struggling in society (“People with mental illness make important contributions to society” and “Living with mental illness has made me a tough survivor”), and one item describes emotion (“I feel comfortable being seen in public with an obviously mentally ill person”). We suggest that all five items on the Stigma Resistance subscale be reworded so that they will be more consistent. Based on our arguments above, we suggest five revised or modified items for Stigma Resistance subscale (see Table S2), and hope future studies can test their psychometric properties.

To explain the less than expected performance of the Stigma Resistance subscale, we proposed two hypotheses: (1) people with different mental illnesses may have different test-retest performances; (2) some items on this subscale might not be stable for more than one month, which resulted in unsatisfactory test-retest reliability.

To test the first hypothesis, we separately analyzed the test-retest reliabilities of Stigma Resistance for four groups of people, which showed an ICC = 0.53 for people with schizophrenia (n = 94), 0.09 for people with depressive disorder (n = 38), 0.88 for people with bipolar disorder (n = 21), and 0.41 for people with anxiety disorder (n = 9). We also used CFA to examine the ISMI theoretical structure on adequately sized samples: schizophrenia (n = 161), and depressive disorder (n = 98). The CFA results showed that people with schizophrenia had better Stigma Resistance factor loadings (first-order standardized factor loadings  = 0.370, 0.851, 0.809, 0.517, and 0.594; second-order standardized factor loading  = 0.226; *ps*<0.05) than did those with depressive disorder (first-order standardized factor loadings  = 0.038, 0.375, 0.928, 0.128, and 0.385; second-order standardized factor loading  = −0.419; *ps*>0.05). Based on these findings, we suspect that the current Stigma Resistance subscale may not be suitable for people with depressive disorder and that it warrants more tests or corroboration. For the second hypothesis, we analyzed each Stigma Resistance item using different time frames, and found that one item (In general, I am able to live life the way I want to) had acceptable test-retest reliability (ICC = 0.73) with an interval <30 days. However, the ICC values (0.27–0.50) of the other four items were unsatisfactory. In addition, Stigma Resistance with a shorter test-retest interval had better reliabilities (ICC = 0.27–0.73 with an interval <30 days;  = 0.29–0.60 for <60 days;  = 0.30–0.53 for <90 days), and we suggest that future research test it within a shorter interval (say, 2 weeks) to validate its test-retest reliability.

Another important finding was that the factor loadings, item intercepts, and construct means were invariant across time. Based on this result, we are confident in concluding that the ISMI can be used to reliably measure the internalized stigma of people with mental illness who are psychologically stable (i.e., respondents with the ability to fill out the ISMI without psychiatric symptoms that interfere with their answers) each time they take the test.

This study has several limitations. First, we do not know any details about how the ISMI was translated and, therefore, are unsure about its linguistic validity. However, we asked two experienced psychiatrists to examine and discuss the wordings of the Taiwan version. In addition, our psychometric properties agree with those reported by Ersoy & Varan [11], Hwang et al. [12], Rensen et al. [14], Ritsher (Boyd) et al. [7], and Sibitz et al. [13]. Therefore, we tentatively conclude that linguistic validity was not a serious problem in this study. Second, all participants were from southern Taiwan, and our results may not be generalizable to the entire population of Taiwan. Third, although participants with various mental illnesses were included in our sample, most had been diagnosed with schizophrenia (45.1%) or depressive disorder (28.2%). Therefore, our results may be more representative of those two types of mental illnesses rather than other kinds of mental illnesses. Additional studies using participants with other kinds of mental illnesses and from other parts of Taiwan are suggested. Fourth, the test-retest interval in this study seemed slightly long, and the internalized stigma of the participants may have changed during such a long period, especially in people with depressive disorder. However, no treatments and interventions were applied to our participants, and our mean test-retest interval (50.23±31.18 days) is comparable to that of Ritsher (Boyd) et al. [7] (6 weeks) and Resen et al. [14] (1 to 3 months). Therefore, our use of the slightly long test-retest interval may somewhat be justified.

In conclusion, the Taiwan version of the ISMI is a valid and reliable instrument for researchers and clinicians who want to measure and evaluate internalized stigma for people with mental illness. Researchers and clinicians can use the ISMI to examine the effects of programs on decreasing internalized stigma for people with mental illness. Moreover, we recommend omitting the Stigma Resistance subscale when using the ISMI.

## Supporting Information

Table S1 **Chinese version of Internalized Stigma of Mental Illness (ISMI) Scale used in this study.** Note: Items are not ordered by each domain.
(DOC)

Table S2 **Suggested items for Stigma Resistance concept.** Note: Future studies are needed to test the psychometric properties of the revised items.
(DOC)
