# Supplementary material for: Psychometric Evaluation of the Internalized Stigma of Mental Illness Scale for Patients with Mental Illnesses: Measurement Invariance across Time
Source: PLoS One. 2014 Jun 2;9(6):e98767. doi: 10.1371/journal.pone.0098767 (PMC4041772; doi:10.1371/journal.pone.0098767)
Supplement: Table S1 — Chinese version of Internalized Stigma of Mental Illness (ISMI) Scale used in this study. Note: Items are not ordered by each domain. (DOC) [file pone.0098767.s001.doc]

Table S1.

| 題目 | 非常不同意 | 不同意 | 同意 | 非常同意 |
| --- | --- | --- | --- | --- |
| 1 因為我有精神疾病，所以這個世界沒有我容身之地。 | 1.□ | 2.□ | 3.□ | 4.□ |
| 2 精神疾病患者是有暴力傾向的。 | 1.□ | 2.□ | 3.□ | 4.□ |
| 3 因為我有精神疾病，所以人們歧視我。 | 1.□ | 2.□ | 3.□ | 4.□ |
| 4 我避免與沒有精神疾病的人接近，以免被他們排斥。 | 1.□ | 2.□ | 3.□ | 4.□ |
| 5 我對於自己有精神疾病感到尷尬或羞愧。 | 1.□ | 2.□ | 3.□ | 4.□ |
| 6 有精神疾病的人不應該結婚。 | 1.□ | 2.□ | 3.□ | 4.□ |
| 7 罹患精神疾病的人也能對社會有重要貢獻。 | 1.□ | 2.□ | 3.□ | 4.□ |
| 8 我覺得自己比不上沒有精神疾病的人。 | 1.□ | 2.□ | 3.□ | 4.□ |
| 9 我不像以前一樣參與社交活動，因為精神疾病可能使我看起來或做起事來比較怪異。 | 1.□ | 2.□ | 3.□ | 4.□ |
| 10 有精神疾病的人無法過好的、有意義的生活。 | 1.□ | 2.□ | 3.□ | 4.□ |
| 11 我不想說太多自己的事，因為我不想讓我的精神疾病造成別人的負擔。 | 1.□ | 2.□ | 3.□ | 4.□ |
| 12 關於精神疾病的負面刻板印象讓我在「正常」世界裡被孤立。 | 1.□ | 2.□ | 3.□ | 4.□ |
| 13 和沒有精神疾病的人在一起，讓我覺得不合適或差人一等。 | 1.□ | 2.□ | 3.□ | 4.□ |
| 14 在公開場合被別人看見我和明顯有精神疾病的人在一起，我覺得輕鬆自在。 | 1.□ | 2.□ | 3.□ | 4.□ |
| 15 只因為我有精神疾病，人們經常對我施以恩惠，或把我當成小孩。 | 1.□ | 2.□ | 3.□ | 4.□ |
| 16 我因為有精神疾病而對自己覺得失望。 | 1.□ | 2.□ | 3.□ | 4.□ |
| 17 罹患精神疾病已經毀了我的生活。 | 1.□ | 2.□ | 3.□ | 4.□ |
| 18 人們可以從我看起來的樣子，分辨出我有精神疾病。 | 1.□ | 2.□ | 3.□ | 4.□ |
| 19 因為我有精神疾病，我需要別人幫我做大部分的決定。 | 1.□ | 2.□ | 3.□ | 4.□ |
| 20 為了避免我家人或朋友尷尬，我遠離社交場合。 | 1.□ | 2.□ | 3.□ | 4.□ |
| 21 沒有精神疾病的人，不可能會了解我。 | 1.□ | 2.□ | 3.□ | 4.□ |
| 22 只是因為我有精神疾病，人們會忽略我或認為我比較不重要。 | 1.□ | 2.□ | 3.□ | 4.□ |
| 23 因為我有精神疾病，我無法為社會貢獻什麼。 | 1.□ | 2.□ | 3.□ | 4.□ |
| 24 與精神疾病共存，使我成為一個堅強的生存者。 | 1.□ | 2.□ | 3.□ | 4.□ |
| 25 因為我有精神疾病，沒有人想和我接近。 | 1.□ | 2.□ | 3.□ | 4.□ |
| 26 整體而言，我可以依照我想要的方式過生活。 | 1.□ | 2.□ | 3.□ | 4.□ |
| 27 儘管我有精神疾病，我還是可以擁有美好而充實的生活。 | 1.□ | 2.□ | 3.□ | 4.□ |
| 28 因為我有精神疾病，其他人認為我在生活中能做成功的事不多。 | 1.□ | 2.□ | 3.□ | 4.□ |
| 29 關於精神疾病的刻板印象，可以適用在我身上。 | 1.□ | 2.□ | 3.□ | 4.□ |
