# Supplementary material for: Psychometric Evaluation of the Internalized Stigma of Mental Illness Scale for Patients with Mental Illnesses: Measurement Invariance across Time
Source: PLoS One. 2014 Jun 2;9(6):e98767. doi: 10.1371/journal.pone.0098767 (PMC4041772; doi:10.1371/journal.pone.0098767)
Supplement: Table S2 — Suggested items for Stigma Resistance concept. Note: Future studies are needed to test the psychometric properties of the revised items. (DOC) [file pone.0098767.s002.doc]

Table S2.

| Subscale | Item description |
| --- | --- |
| *Stigma*  *Resistance* | I feel comfortable being seen as an obviously mentally ill person |
|  | I am able to live life the way I want to, despite my mental illness |
|  | I can have a good, fulfilling life, despite my mental illness |
|  | I can make important contributions to society, despite my mental illness |
|  | I have become a tough survivor because of living with mental illness |
